# Surface Nanotexturing of Boron-Doped Diamond Films by Ultrashort Laser Pulses

**DOI:** 10.3390/mi14020389

**Published:** 2023-02-04

**Authors:** Matteo Mastellone, Eleonora Bolli, Veronica Valentini, Stefano Orlando, Antonio Lettino, Riccardo Polini, Josephus Gerardus Buijnsters, Alessandro Bellucci, Daniele Maria Trucchi

**Affiliations:** 1 CNR-ISM, DiaTHEMA Lab, U.O.S. Montelibretti, Via Salaria km 29.300, 00015 Monterotondo, Italy; 2 CNR-ISM, FemtoLAB, U.O.S. Tito Scalo, Zona Industriale, 85050 Tito, Italy; 3 Dipartimento di Scienze e Tecnologie Chimiche, Università di Roma ‘Tor Vergata’, 00133 Rome, Italy; 4 CNR-IMAA, Zona Industriale, 85050 Tito, Italy; 5 Department of Precision and Microsystems Engineering, Delft University of Technology, Mekelweg 2, 2628 CD Delft, The Netherlands

**Keywords:** boron-doped diamond, laser texturing, ripples, LIPSS, surface functionalization

## Abstract

Polycrystalline boron-doped diamond (BDD) films were surface nanotextured by femtosecond pulsed laser irradiation (100 fs duration, 800 nm wavelength, 1.44 J cm^−2^ single pulse fluence) to analyse the evolution of induced alterations on the surface morphology and structural properties. The aim was to identify the occurrence of laser-induced periodic surface structures (LIPSS) as a function of the number of pulses released on the unit area. Micro-Raman spectroscopy pointed out an increase in the graphite surface content of the films following the laser irradiation due to the formation of ordered carbon sites with respect to the pristine sample. SEM and AFM surface morphology studies allowed the determination of two different types of surface patterning: narrow but highly irregular ripples without a definite spatial periodicity or long-range order for irradiations with relatively low accumulated fluences (<14.4 J cm^−2^) and coarse but highly regular LIPSS with a spatial periodicity of approximately 630 nm ± 30 nm for higher fluences up to 230.4 J cm^−2^.

## 1. Introduction

Boron-doped diamond (BDD) is a material of interest for many applications such as electrochemistry, bio-sensing, and microelectromechanical systems [1,2]. BDD is an excellent electrode material with a wide potential window in liquid solution, low background current, high thermal and chemical stability, and a long lifetime, which makes it particularly suitable for electro-catalytic processes [3]. However, even if diamond can efficiently emit electrons in an aqueous environment (i.e., as solvated electrons [4]) when the surface is properly functionalized (e.g., with hydrogen termination to achieve negative electron affinity), it requires UV radiation to promote electrons in the conduction band due to its large bandgap (5.5 eV). For this reason, diamond cannot be directly used to harvest and convert solar radiation. With the aim of improving its electrochemical properties, the nanostructuring of the diamond surface is one of the more explored approaches for enhancing important features such as sensitivity and selectivity to chemical reactions, and electric transport by modifying the surface chemistry [5]. Among the different methods used for achieving nanostructured surfaces for BDD [6], the use of pulsed lasers to fabricate laser-induced periodic surface structures (LIPSS) was introduced some years ago [7], demonstrating the potential capability for altering the surface morphology and tuning the electrochemical properties of BDD plates. The induced morphological and chemical alterations are strictly correlated to the impinging laser beam properties. In the past, our group showed that even in the absence of post-irradiation processing, it is possible to structurally alter diamond by inducing mechanical stresses that can be assessed by Raman characterization [8]. It is widely known, in fact, that mechanical strains can alter the electronic, optical, and mechanical properties of 2D crystals [9] and that “elastic strain engineering” is becoming a useful tool for bulk materials as well [10]. On the other hand, chemical modifications can be laser-induced (e.g., superficial oxidation [11]), or new chemical functional groups can be introduced post-irradiation via surface adsorption [12]. In this context, characterization by Raman spectroscopy is useful for assessing possible chemical modifications.

The ability to absorb visible radiation was demonstrated on different types of diamond [8,13], enabling the material for employment in a wide range of applications [14]. According to these considerations, BDD plates with LIPSS can be used as efficient solar photo-electrodes that are able to absorb solar radiation on one side, promote photo-charge carriers, and be the reactive site for selective chemical reactions on the other side.

However, no systematic study has been reported so far on the effect of the key laser parameter on LIPPS formation on BDD (e.g., pulse energy, accumulated fluence). Indeed, the analysis of structural modifications characterizing LIPSS such as periodicity, depth, and chemical structure is essential in understanding how to tune this material for specific applications and modify the morphology according to defined requirements.

In this paper, the effect of the accumulated laser fluence, varied by changing the number of pulses at a fixed pulse energy, is analysed to evaluate its role in BDD surface nanotexturing.

## 2. Materials and Methods

### 2.1. Diamond Growth

Polycrystalline BDD films (4-μm thick) were deposited on a silicon wafer by a hot-filament chemical vapor deposition (HF-CVD) reactor (sp^3^ Diamond Technologies, Santa Clara, CA, USA), following a seeding by detonation diamond nanoparticles [15]. The growth conditions were: 2.4% CH_4_/H_2_ gas ratio (72 sccm/3000 sccm), 40 sccm trimethylborane (TMB), and substrate temperature of ~850 °C. The achieved B-doping level was about 2.8 at.% [7], and the BDD film resistivity was around 5 × 10^−3^ Ω cm. For this study, the samples were created by dicing the wafer into pieces of 1.5 × 1.5 cm^2^.

### 2.2. Laser Texturing

The surface nanotexturing treatments were performed using a Ti:Sapphire femtosecond laser (Spectra-Physics, Milpitas, CA, USA) with the following characteristics: 100 fs pulse duration, linear polarization, wavelength *λ* = 800 nm, adjustable repetition rate up to 1 kHz. The laser beam was generated by a mode-locked oscillator and subsequently regeneratively amplified. The beam was focused perpendicularly on the surface of the sample and positioned within a vacuum chamber at a pressure <5 × 10^−7^ mbar to minimize sample oxidation or nitridation. Spot-size was assessed a posteriori by analysing the ablation and modification areas with scanning electron microscopy (SEM).

### 2.3. Structure and Morphology Characterization

Surface morphology was analysed by Field-Emission Gun Scanning Electron Microscopy (FEG-SEM) obtained by a Zeiss Microscope (model Leo Supra 35; Oberkochen, Germany) and by Atomic Force Microscopy (AFM) investigations conducted using an OmegaScope platform (HORIBA Ltd., Kyoto, Japan). AFM imaging was performed in tapping mode, setting the operational amplitude at 50 nm, and using a silicon pyramidal tip (MikroMasch HQ:NSC14/Al BS; Wetzlar, Germany) with a characteristic radius of ~8 nm, a height of ~15 µm, and a resonance frequency of 137 kHz. The scan rate was fixed at 0.2 Hz. All the AFM data were acquired, filtered, and analysed using the AIST-NT SPM control software.

The software Gwyddion (version 2.62) was used to perform 2D Fast Fourier Transformation (2D-FFT) analysis of 30 × 30 µm^2^ sized SEM images to obtain reliable values of LIPSS periodicity.

Structural investigations were carried by Raman spectroscopy using a Horiba Scientific LabRam HR Evolution confocal spectrometer (HORIBA Ltd., Kyoto, Japan) equipped with a 100 mW Oxxius laser source (wavelength of 532 nm), a computerized XY-table, an electron-multiplier CCD detector, and an Olympus U5RE2 (Tokyo, Japan) microscope with a 100× objective (laser spot on the sample surface 0.7 μm) with a numerical aperture (NA) of 0.9 and a grating with 600 grooves/mm. All Raman spectra were recorded with backscattering geometry focusing 10 mW at the sample, and two spectra with an accumulation time of 100 s were averaged.

## 3. Results and Discussion

In this section, we report on the analysis of morphological and structural properties of the irradiated samples. The work was carried out by impinging single spots on the BDD surfaces (e.g., absence of relative movement), aimed at analysing the effect of laser treatments on the resulting morphological and structural properties after a specific number of pulses.

Table 1 summarizes the relevant experimental parameters for the studied treatments; all parameters were obtained by applying a fixed pulse energy, *E_p_* = 0.65 mJ/pulse. Preliminary experiments were carried out with higher pulse energies, but the material was severely damaged even when impinged with a low number of pulses (<10). For this reason, the pulse energy selected was the highest that allowed for the absence of unwanted morphological alterations (such as pits and/or delamination) at the centre of the spot, where the intensity is higher, up to 150 pulses.

The produced spots have different accumulated fluence, *Φ_A_*, defined as *N* × *Φ_P_*, where *N* is the number of laser pulses released on the same spot and *Φ_P_* is the single pulse fluence equal to *E_P_*/*(πr*^2^*)*, where *r* is the radius of the Gaussian beam circular section on the focal plane. The different samples are denoted by the acronym “*PN*” to indicate the total number of laser pulses per spot (with *N* varying from 1 to 160).

### 3.1. Morphological Analysis of Irradiated Spots

#### 3.1.1. SEM Characterization

Preliminary SEM investigations were performed to calculate the diameter of the circular spot on the focal plane of the Gaussian beam profile, *w*. This value was estimated to be approximately 120 µm by analysing the ablation and modification areas and by applying the method proposed by Liu [16]. All the SEM images reported in this work were taken at the centre of the laser spot. Since the laser beam has a Gaussian irradiance distribution, the periphery of the spot is irradiated by an energy intensity that is lower than the modification threshold for LIPSS fabrication. However, as the treatments are commonly performed on a larger area rather than on single spots for applications, this unwanted effect would not pose a problem since the superimposition of multiple laser pulses during the translational movements (with irradiation steps impinging the below-threshold crown) would in effect not leave low, accumulated fluence borders.

**Figure 1:**
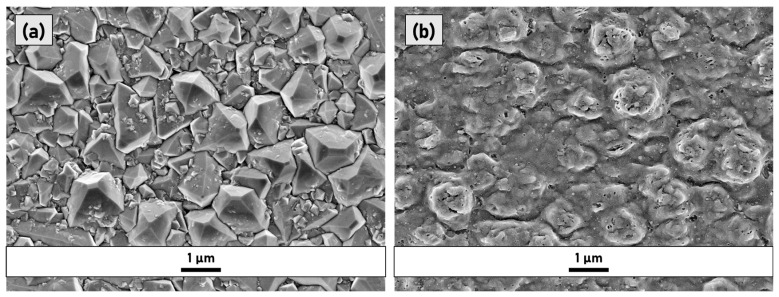
SEM micrographs of (**a**) the pristine (untreated) sample and (**b**) the sample P1.

Figure 1 shows SEM micrographs of the sample surface prior to and after irradiation with a single laser pulse. As expected, since LIPSS formation is a multi-pulse phenomenon [17], the presence of periodic structures was not observed after the first irradiation; however, solely localized ablation was observed due to inhomogeneous energy deposition. The induced surface roughness at the nanoscale acts as a “seed” giving origin to the surface scattered field which consequently interferes with the incident beam, and thus promotes the formation of LIPSS [17].

**Figure 2:**
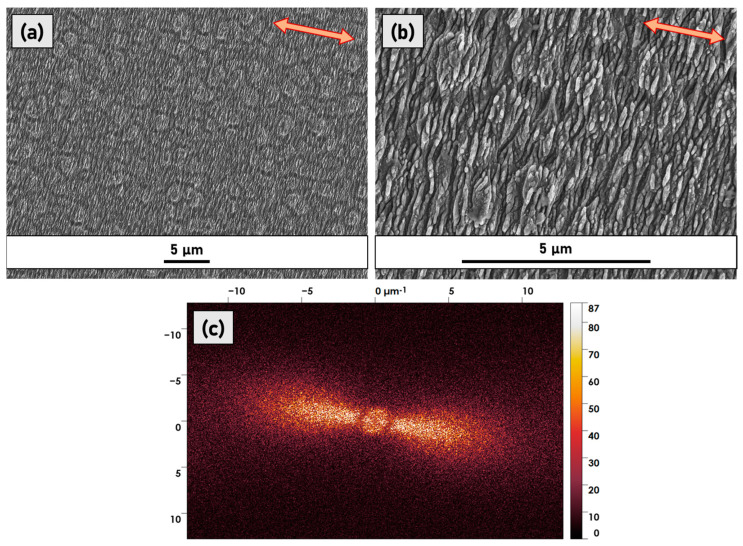
SEM micrographs of sample P5 at (**a**) low magnification and (**b**) high magnification. The red arrows indicate the laser polarisation direction. In (**c**) the 2D−FFT spectrum is shown obtained by analysing the SEM micrograph reported in (**a**).

Figure 2 shows the SEM images of sample P5. Panels (a) and (b) reveal that the laser treatment induced the formation of irregularly shaped and unevenly distributed ripples running along the axis perpendicular to the laser polarisation direction in a grating-like pattern. Such ripples are not coherently linked, and at best, they are a few micrometres long. They do not present a periodic pattern, allowing us to exclude categorization of the texturing as LIPSS. The results are confirmed by the 2D-FFT spectrum displayed in Figure 2c, which provides two-dimensional histograms of the spatial frequencies from the original SEM micrographs [18]. A conventional unidimensional LIPSS pattern should present a 2D-FFT spectrum consisting of sickle-shaped features for which the centre position determines the most frequent spatial period, *Λ*. The “sickle” width, on the other hand, quantifies the distribution of the spatial frequencies. In the 2D-FFT spectrum presented in Figure 2c, the expected sickle-shaped figure is replaced by a pear-shaped distribution suggesting the presence of many spatial periodicities (spanning from 100 to 700 nm) without a dominant one and an increasing opening angle that serves as a measure of angular deviation from the ideally ordered LIPSS grating [18].

**Figure 3:**
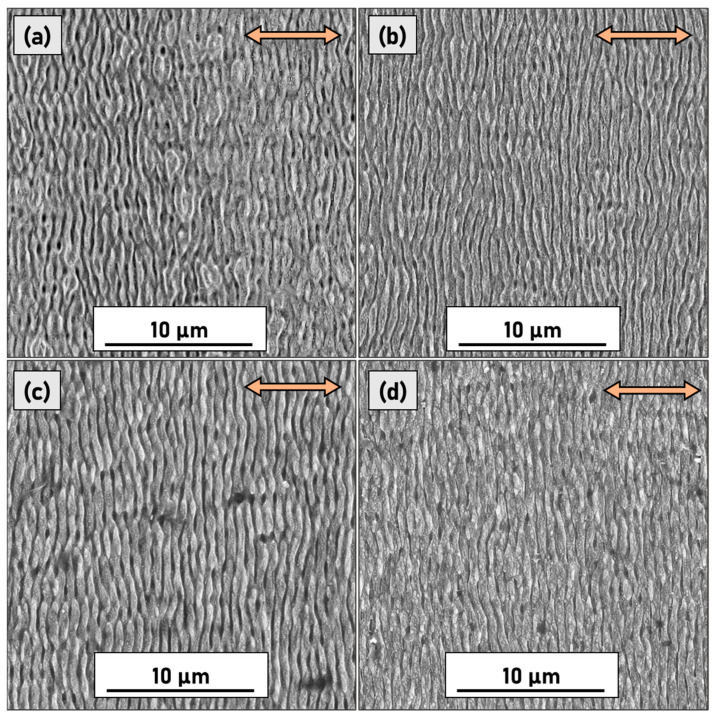
SEM micrographs of (**a**) P10, (**b**) P30, (**c**) P50, and (**d**) P100 samples. The red arrows indicate the laser polarisation direction.

SEM images taken at the same magnification (2.5 k×) for samples P10, P30, P50, and P100 are shown in Figure 3. For all the samples treated with accumulated fluences in the range spanning from 14.4 to 144 J cm^−2^, the LIPSS patterning is clearly discernible. The spatial periodicity of the unidimensional LIPSS was calculated using the 2D-FFT technique and was evaluated to be approximately 630 nm (the uncertainty of the average spatial periodicity is at its lowest, i.e., 30 nm for P50); thus, the LIPSS can be categorized as Low Spatial Frequency LIPSS (LSFL),.i.e., unidimensional patterns with spatial periodicity close to the laser wavelength and, more precisely, in the range *λ*/2 < *Λ* ≤ *λ*.

The physical mechanisms that govern the formation of LIPSS are still debated in literature; nevertheless, for LIPSS generated by pulses in the femtosecond range, the electromagnetic theory that links their formation to the interference of the incident beam with a surface scattered electromagnetic wave (SEW) is the most accepted [19]. SEWs are usually generated by scattering of the incident laser beam at the surface nanoroughness.

The interference between the incoming laser beam and SEWs induces a periodic energy deposition that triggers localized ablation. The resulting morphology is a succession of peaks and valleys with a definite spatial periodicity. For highly absorbing materials such as metals, the surface scattered field is an excited surface plasmon polariton [17], and its interference with the laser light produces spatial periodicities close to the laser wavelength. On the other hand, LIPSS on dielectrics are originated by coherent superposition between the scattered near-field at the surface and the incident electromagnetic field produced by the propagating beam and usually present *Λ* < *λ*/2 [20,21].

The reported LIPSS pattern (see Figure 3) is morphologically more akin to the one usually found on highly absorbing materials. It is important to emphasize that the used samples were highly doped, and therefore, unlike intrinsic diamond, they are not blind to the used laser wavelength of 800 nm. Consequently, their interaction with the laser beam can be comparable to highly absorbing semiconductors (such as silicon) that temporarily turn into a metallic state during the irradiation [22,23].

SEM images also show that the long-range periodicity and homogeneity of the texturing improve as the number of pulses increases. The irregularity of LIPSS is prominent in the micrograph of sample P10 (Figure 3a), in which it is possible to discern the wavy nature of the grooves, bifurcations, and in general, an uninterrupted length that extends for just a few micrometres.

**Figure 4:**
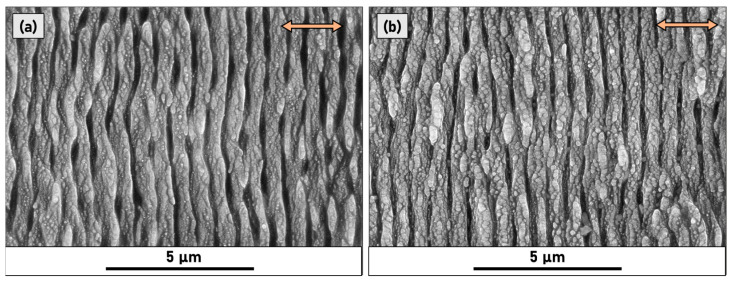
SEM micrographs of (**a**) sample P50 and (**b**) sample P160. The red arrows indicate the laser polarisation direction.

As the number of pulses increases, the long-range periodicity improves due to positive feedback caused by the deepening of the grooves [24], and consequently a better coupling between laser light and scattered surface waves. At the same time, the increasing accumulated fluence has another effect; namely, the formation of irregular nanostructures on the surface of ripples at a higher hierarchical level of LIPSS. This phenomenon is easily visible in Figure 4 where samples P50 and P160 are compared at a higher magnification.

**Figure 5:**
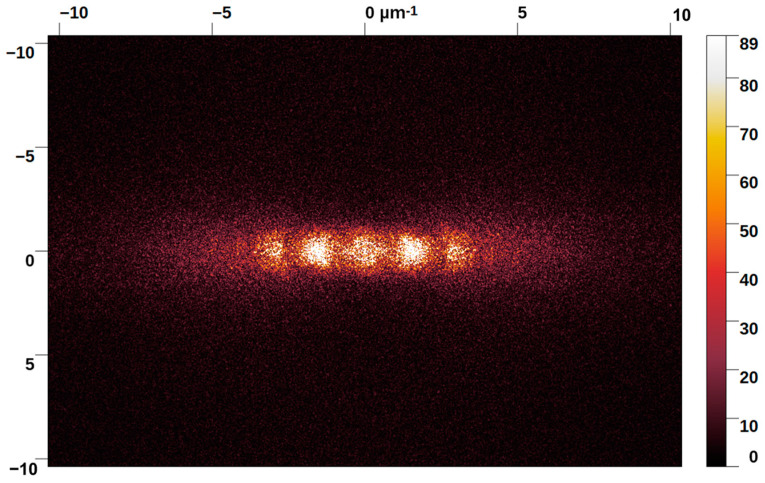
2D−FFT spectrum obtained by analysing a 30 × 30 μm^2^ SEM micrograph of sample P50.

The emergence of nanostructure in higher hierarchical levels was demonstrated in metals such as steel [25] and cobalt [26]; consequently, this finding is beneficial for the enhancement of optical absorption [27]. The 2D-FFT spectrum of sample P50 is shown in Figure 5 where a spatial periodicity of 630 ± 30 nm was calculated. In P50, the LIPSS patterning presents the best long-range homogeneity, as assessed by 2D-FFT. This sample presents the lowest “sickle” width and opening angle of all the analysed samples.

Indeed, it was not possible to extract a meaningful trend of the opening angle and “sickle” width as a function of the pulse number due to the “noisy” nature of the 2D-FFT spectrum obtained, which had a rather large uncertainty in spatial periods displayed by the obtained 1D-patterning. The broadness of the 2D-FFT spectrum could be related to the high roughness of the starting sample. It is important to point out that in literature it has been reported that LIPSS periodicities can be correlated to the number of impinging pulses, where the periodicity tends to decrease as the number of pulses increase [28].

#### 3.1.2. AFM Characterisation

A high-resolution AFM investigation was performed to derive data on the third dimension, i.e., depth of the LIPSS, due to its importance in the alteration of physical properties such as optical absorption. Due to the formation of irregular structures in samples treated at low accumulated fluence and the high roughness of the starting surface, it was not possible to validate a significant trend for the depth as a function of the pulse number.

**Figure 6:**
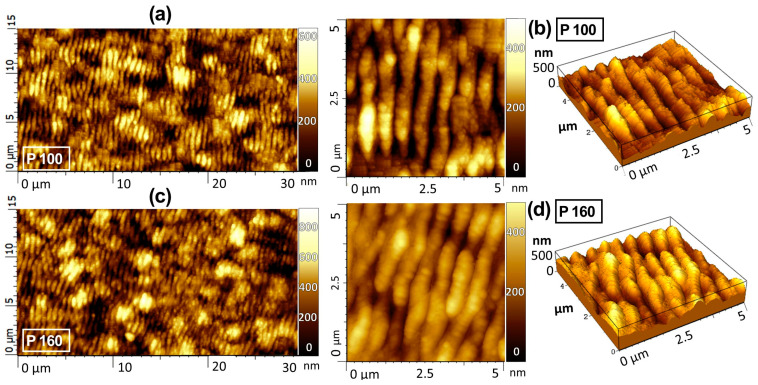
AFM images of sample P100: (**a**) map of 15 × 30 μm^2^; (**b**) map of 5 × 5 μm^2^ (shown in both top-view and tilted-view representation), and sample P160: (**c**) map of 15 × 30 μm^2^ and (**d**) map of 5 × 5 μm^2^ (shown in both top-view and tilted-view representation).

**Figure 7:**
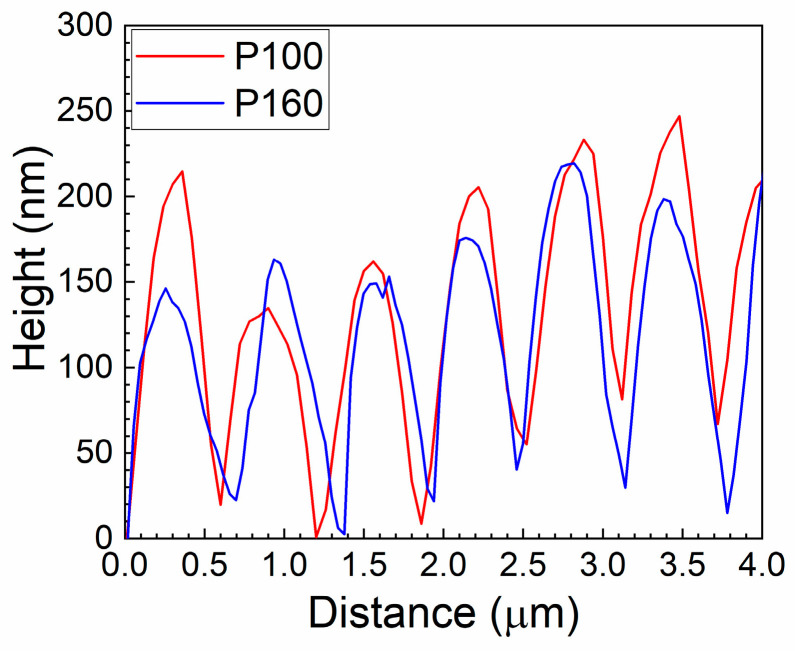
AFM line profiles taken from samples P100 and P160 along a line of 4 µm taken at the centre of the images shown in Figure 6b,d, respectively.

Figure 6 shows the topographic AFM images for the P100 and P160 samples. The AFM topography confirms the regularity of the formed LSFL periodicity as well as the almost constant pattern peak-to-valley depth of 150 ± 20 nm for samples P100 and P160. The corresponding line height profiles taken from both samples (see Figure 7) further confirm this observation.

**Figure 8:**
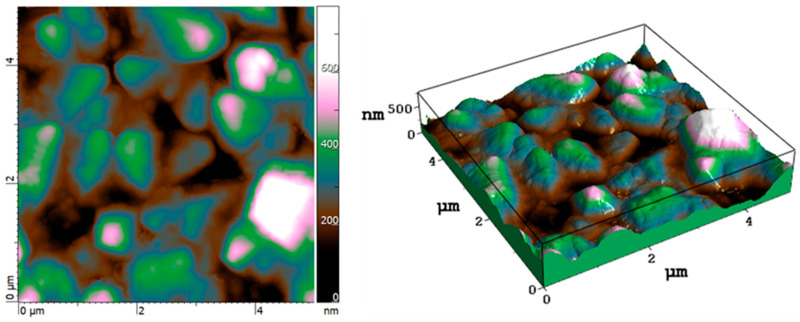
Top-view and tilted-view topographic AFM images (5 × 5 μm^2^) of the untreated BDD sample surface.

We found average values of 120 ± 40 nm and 135 ± 50 nm for P30 and P50, respectively, although they presented a higher dispersion of depth (with a measurement error larger than 30% within the profiles) if compared to P100 and P160 samples. This uncertainty does not allow a reliable assessment of the tendency of the depth for such experiments. Nevertheless, the laser irradiation on a BDD surface was still able to produce LIPSS, characterized by the typical large and randomly oriented grains of the polycrystalline diamond growth (Figure 8) [29]. Calculations of the roughness parameters (the roughness average, R_a_, and the root mean square roughness, R_ms_) made on a sampling area of 25 µm^2^ reveal that the laser treatment transformed the surface roughness from being randomly distributed to being regularly patterned, evident with an approximate 30% decrease in R_a_ and R_ms_ (R_a_ of 113.4 ± 5.0 nm and 77.3 ± 5.0 nm, R_ms_ of 87.5 ± 5.0 nm and 61.4 ± 4.5 nm, for the untreated and the treated samples, respectively).

### 3.2. Structural Characterisation of Irradiated Spots

**Figure 9:**
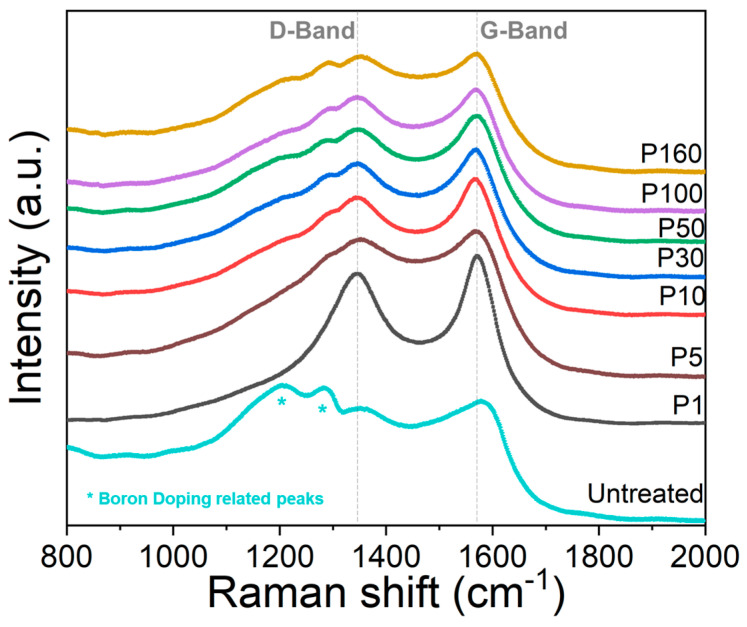
Raman spectra of all the samples listed in Table 1, together with the spectrum of the untreated sample. D band at ~1360 cm^−1^ and G band at ~1590 cm^−1^ are highlighted with dotted grey lines.

Figure 9 shows the Raman spectra for all the different samples reported in Table 1, together with the untreated sample, in the range 800–2000 cm^−1^. All the characterization was performed at the centre of the spot. In these spectra, it is possible to note an evolution of the contributions of the D band and G band as a function of the number of pulses. Generally, the laser treatment induces an enhanced graphitisation and/or disorder in the BDD sample, the effect of which only varies negligibly as the number of pulses is over five.

By comparing the spectrum of the untreated sample with respect to those of treated ones at the different accumulated fluences, it is possible to note that the two bands (highlighted with the asterisk symbol) between 1200 and 1300 cm^−1^, typical of the heavily boron-doped diamond [30,31] but different to the one-phonon line located at 1332 cm^−1^ for undoped (or lightly doped) diamond, disappear after the laser treatment. Conversely, the contribution of the D band (~1360 cm^−1^) and G band (~1590 cm^−1^) becomes significantly higher after the laser treatment, especially just after one single pulse. Moreover, a slight variation of the D and G bands’ positions (down to ~1345 cm^−1^ and ~1575 cm^−1^, respectively) is observable for the treated samples, occurring when the sp^2^ disorder increases. However, this transition is far from the carbon amorphization, and the G band derives effectively from the presence of graphite contributions [32]. On the other hand, the I_D_/I_G_ ratio decreases from 0.96 for the untreated sample to 0.61 ± 0.05 for the longest treated samples, thus further indicating an evolution towards order for the graphitic components of the samples. Finally, it is possible to state that samples treated at different values of accumulated fluence display the same chemical composition regardless of the morphology. In addition, even the minimum accumulated fluence (1.44 J/cm^2^, corresponding to one single irradiation pulse) enhances the sp^2^ film contribution by increasing the graphite content probably at the grain boundaries, although no evidence of nanostructure formation has been found.

## 4. Conclusions

Laser treatments are effective in producing a surface periodic structure on polycrystalline BDD, with the formation of regular ripples once a certain value of fluence (14.4 J/cm^2^) is reached. Under this threshold, irregular periodic structures are formed with a period spread from 100 to 700 nm. By increasing the accumulated laser fluence, it is possible to vary the morphology of the BDD surface to obtain LFSL with periodicity of 630 nm, which is an attractive value corresponding to visible radiation wavelength. Considering the enhanced optical properties, which have already been reported for the intrinsic diamond samples in the visible-IR range [33,34], this could allow for an efficient implementation of BDD plates as photo-electrodes stimulated by solar radiation. On the other hand, preliminary basic electrochemical characterization indicated that the induction of irregular HSFL on BDD samples is beneficial to the improvement of functional properties [7]. In this direction, the use of an alternative technique to achieve deep-subwavelength 2D periodic surfaces [35], that presents a reduced periodicity but higher regularity, could improve the performance. Additionally, by considering the results of AFM and Raman spectroscopy obtained in this study, which highlighted an average depth of the LIPSS of about 150 nm and the formation of a distinct sp^2^ graphitic layer over the BDD film, an alternative potential application for LIPSS on this material could be implementation as the THz component by exploiting the anisotropy of the formed metal/dielectric patterns [36].

## Figures and Tables

**Table 1:** Experimental parameters of the seven different laser irradiation treatments.

Sample	Single Pulse Energy (mJ)	Single Pulse Fluence (J cm^−2^)	No. of Pulses per Spot	Accumulated Laser Fluence (J cm^−2^)
P1	0.65	1.44	1	1.44
P5	0.65	1.44	5	7.20
P10	0.65	1.44	10	14.4
P30	0.65	1.44	30	43.2
P50	0.65	1.44	50	72.0
P100	0.65	1.44	100	144
P160	0.65	1.44	160	230

## Data Availability

Not applicable.
